# Wilson’s Dissector

**Published:** 1844-01

**Authors:** 


### WILSON'S DISSECTOR.

To the same prolific press the student of anatomy is indebted for another aid in the prosecution of this important study. Wilson's Anatomy has been much admired for its accurate and beautiful delineations, and the present work lays claim to a portion of the same excellence. It has been arranged to suit medical students in the United States, by Paul B. Goddard, M.D., Demonstrator of Anatomy in the University of Pennsylvania. It strikes us as being all that a "Dissector" could be. The wood cuts are numerous, and will afford the student the most essential aid in the dissecting room. Y.

